# Prognostic significance of incident atrial fibrillation following STEMI depends on the timing of atrial fibrillation

**DOI:** 10.1007/s12471-015-0709-2

**Published:** 2015-05-29

**Authors:** P. Gal, E. Parlak, F. Demirel, A. Adiyaman, J. ten Berg, A.W.J. van ’t Hof, A. Elvan

**Affiliations:** 1 Cardiology Departments, Isala, Zwolle, The Netherlands; 2 St Antonius Hospital, Nieuwegein, The Netherlands; 3 Isala Klinieken, Dr. Van Heesweg 2, 8025 AB Zwolle, The Netherlands

**Keywords:** Atrial fibrillation, STEMI, Mortality, Age, Killip class

## Abstract

Atrial fibrillation (AF) is associated with short-term mortality after ST-elevation myocardial infarction (STEMI), but there is limited data on the temporal association between AF and mortality after STEMI. A total of 830 patients were included (age: 62 ± 12 years, 76 % male). Patients with new-onset AF < 30 days after STEMI were divided among three subgroups: AF on the day of admission, AF 24–72 h and AF > 72 h after admission. Thirty-day mortality was assessed by telephone and via the municipal population registry. Twenty patients died < 30 days after admission. In 41 patients, AF was detected on the day of admission, in 14 patients 24–72 h after admission and in 18 patients > 72 h after admission. Mortality was higher in patients with AF on the day of admission (7.3 vs 2.2 %, *p* = 0.036) and 24–72 h after admission (14.3 vs 1.4 %, *p* < 0.001), but not in patients with AF > 72 h after admission (0 vs 1.1 %, *p* > 0.999). Age (odds ratio (OR) 1.123, *p* < 0.001), Killip class (adjusted OR 8.341, *p* < 0.001), AF on the day of admission (OR 3.585, *p* = 0.049) and 24–72 h after admission (OR 11.515, *p* = 0.003) were, amongst other variables, associated with an increased 30-day mortality. In conclusion, only new-onset incident AF during the first 72 h after admission was associated with 30-day mortality in STEMI patients.

**Electronic supplementary material:**

The online version of this article (doi:10.1007/s12471-015-0709-2) contains supplementary material, which is available to authorized users.

## Introduction

Atrial fibrillation (AF) is common in the setting of ST-elevation myocardial infarction (STEMI) [1–4] and is associated with an increased re-infarction rate, cardiogenic shock and pulmonary oedema [5]. AF is also an independent predictor of both short-[6, 7] and long-term mortality [5, 7–12]. However, there are only limited data on the temporal association between new-onset AF and short-term mortality. The present study was aimed at assessing the impact of different AF episode timings on 30-day mortality in patients who underwent primary percutaneous coronary intervention (PPCI) in the setting of STEMI.

## Methods

Our population consisted of patients with a STEMI, who were admitted for PPCI and included in the ongoing tirofiban in myocardial infarction evaluation (On-TIME) II study [13, 14], a prospective, multicentre, placebo-controlled, randomised, clinical trial. The rationale, design and primary results of On-TIME II have been previously described [13]. Briefly, enrolment was from June 2006 to November 2007. Eligible patients were aged 21–85 years with symptoms of acute myocardial infarction of more than 30 min but less than 24 h and ST-segment elevation of more than 1 mV in two adjacent electrocardiograph (ECG) leads. Exclusion criteria were severe renal dysfunction (glomerular filtration rate < 30 ml/min or serum creatinine > 200 mmol/l (> 2.5 mg/dl)), therapy resistant cardiogenic shock (systolic blood pressure ≤ 80 mmHg for > 30 min), persistent severe hypertension (systolic pressure > 180 mmHg or diastolic pressure > 110 mmHg), or a contraindication to anticoagulation or increased risk of bleeding. Also, patients with a left bundle branch block, pregnant and/or breastfeeding women, and patients with a life expectancy of less than 1 year were excluded. For the present study, patients with a known history of AF were excluded. From each patient, written informed consent for participation in the On-TIME II study was obtained. The local ethics committees approved the study protocol.

## Treatment

All patients were planned to undergo PPCI and were treated according to the On-TIME II study protocol, randomly assigned to (prehospital) treatment with tirofiban (25 μg/kg bolus and 0.15 μg/kg/min maintenance infusion for 18 h) or placebo. PPCI was performed with standard techniques, if the coronary anatomy was suitable for angioplasty. Additional treatment with stents and devices was at the discretion of the treating cardiologist. All patients were treated with optimal drug therapy including angiotensin-converting enzyme inhibitors, β-blockers, aspirin and a statin. Final discharge and admission duration was at the discretion of the treating cardiologist.

## Endpoints

The primary endpoint of the On-TIME II trial was the extent of residual ST-segment deviation at 1 h after PCI. For the present study, all-cause mortality was the primary endpoint of this study. To assess mortality, all patients visited the outpatient clinic 30 days after admission. When patients did not visit the outpatient clinic, an attempt was made to contact the patient and general practitioner to determine whether the patient was still alive. Furthermore, the municipal population registries, which keep records of all deaths in participating countries, were checked in all patients > 30 days after the STEMI. The occurrence of AF was defined as an episode of AF/atrial flutter/atrial tachycardia > 30 s, either on a telemetry strip or on a 12-lead ECG, in accordance with European guidelines [15] within 30 days after admission. Patients were on telemetry during the first 48 h after admission, and an ECG was performed once every 24 h, or whenever deemed necessary. Whenever a patient experienced symptoms and was not on telemetry, an ECG was performed immediately.

### Statistical analysis

**Fig. 1:**
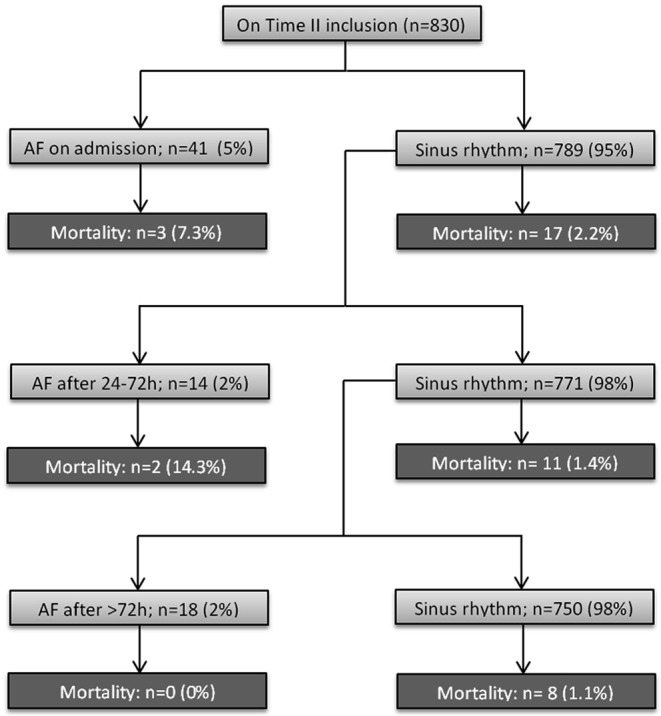
Flow chart of patient categorisation into AF subgroups and mortality. Patient categorisation flow chart. *AF* atrial fibrillation

Continuous variables were expressed as mean with standard deviation (SD). Patients in whom AF was detected < 24 h after admission were categorised to the ‘AF on the day of admission’ subgroup. Patients in whom AF was detected 24–72 h after admission were categorised to the ‘AF 24–72 h after admission’ subgroup. Patients in whom AF was detected > 72 h but < 30 days after admission were categorised to the ‘AF > 72 h after admission’ subgroup, as displayed in Fig. 1. Baseline characteristics between 30-day mortality yes and no groups were compared with a Mann–Whitney *U* test in case of continuous variables and chi-square test in case of dichotomous or categorical data, except for data with > 20 % cells with a value < 5, in which case a Fisher’s exact test was used. A univariate analysis was performed for the association between patient characteristics, biomarkers, AF and mortality with a binary logistic regression model. A multivariate model was not created due to the limited number of endpoints in this study. However, for each AF episode timing, we performed a multivariate analysis with the Zwolle risk score [16]. The Zwolle risk score is a composite score of 6 clinical variables: Killip class, TIMI flow post-PPCI, age, 3-vessel disease, anterior infarction and ischaemia time, and allows identification of STEMI patients at high risk of 30-day mortality. The difference between patient age in smoking versus non-smoking patients was assessed with a Mann–Whitney *U* test. Statistical analysis was performed using IBM SPSS statistics version 20 (IBM inc., Armonk, NY, USA). A *p*-value of ≤ 0.05 was considered statistically significant.

## Results

A total of 984 patients were admitted with the diagnosis of STEMI. Of these patients, 861 underwent PPCI. Baseline characteristics of the whole study population have been previously reported [14]. A total of 31 patients had a history of AF and were subsequently excluded; 30-day follow-up was present in 830 patients, who were included in the analysis for the current study. Baseline characteristics of the study population are displayed in supplemental Table 1.

## Mortality and baseline characteristics

A total of 20 patients died < 30 days after STEMI, 5 patients died < 24 h after admission, 3 patients died 24–72 h after admission and 12 patients died > 72 h after admission. AF was detected in a total of 73 patients: in 41 patients on the day of admission, in 14 patients 24–72 h after admission and in 18 patients > 72 h after admission, as displayed in Fig. 1. Patients who died were significantly older (74.2 vs 61.8 years, *p* < 0.001), less often smokers (21.1 vs 49.3 %, *p* = 0.015), with more often a history of diabetes (35.0 vs 10.1 %, *p* < 0.001) and hypertension (55.0 vs 32.7 %, *p* = 0.037), more often a Killip class > 1 (50.0 vs 10.7 %, *p* < 0.001), more often a previous infarction (25.0 vs 7.8 %, *p* = 0.006), a lower systolic (109.1 vs 131.4 mmHg, *p* < 0.001) and diastolic blood pressure on admission (63.3 vs 76.9 mmHg, *p* = 0.001), received the tirofiban study medication less often (20.0 vs 49.6 %, *p* = 0.009) and more often a TIMI grade flow < 3 post PPCI (40.0 vs 8.2 %, *p* < 0.001), as displayed in supplemental Table 1.

## Atrial fibrillation on day of admission

Mortality in patients in whom AF was detected on the day of admission was significantly higher compared with patients who were AF-free (7.3 vs 2.2 %, *p* = 0.036), as displayed in Table 1. In univariate analysis, age (odds ratio (OR) 1.123, *p* < 0.001), smoking (OR 0.275, *p* = 0.023), diabetes (OR 4.774, *p* = 0.001), hypertension (OR 2.514, *p* = 0.043), Killip class > 1 (OR 8.314, *p* < 0.001), previous myocardial infarction (OR 3.942, *p* = 0.010), systolic (OR 0.641, *p* < 0.001) and diastolic (OR 0.530, *p* < 0.001) blood pressure on admission, tirofiban study medication (OR 0.254, *p* = 0.015) and TIMI grade post PPCI < 3 (OR 7.444, *p* < 0.001) were associated with 30-day mortality. AF on the day of admission was also significantly associated with 30-day mortality (OR 3.585, *p* = 0.049). In multivariate analysis, AF on the day of admission was not associated with short-term mortality (adjusted OR 3.537, *p* = 0.116). The analysis is displayed in Table 2.

**Table 1:** Association between 30-day post-infarction mortality and atrial fibrillation

	Mortality		
	AF free	AF	*p*-value
AF on the day of admission	17/789 (2.2 %)	3/41 (7.3 %)	0.036
AF 24–72 h after admission	11/771 (1.4 %)	2/14 (14.3 %)	< 0.001
AF > 72 after admission	8/750 (1.1 %)	0/18 (0 %)	> 0.999

Data are presented as absolute number with percentages. *p*-value of rejecting the null hypothesis of no relationship between AF and mortality

**Table 2:** Odds ratio analysis for all-cause 30-day post-infarction mortality

Univariate	OR	95 % CI	*p*-value
Age (per year)	1.123	1.065–1.184	< 0.001
Gender male	1.733	0.682–4.406	0.248
BMI	1.035	0.901–1.188	0.628
Current smoker	0.275	0.090–0.835	0.023
Diabetes	4.774	1.852–12.304	0.001
Hypertension	2.514	1.029–6.140	0.043
Hypercholesterolaemia	0.957	0.344–2.665	0.933
Killip class > 1	8.314	3.365–20.544	< 0.001
Previous MI	3.942	1.387–11.200	0.010
Previous PCI	0.584	0.077–4.428	0.603
Systolic BP (per 10 mmHg)	0.641	0.516–0.796	< 0.001
Diastolic BP (per 10 mmHg)	0.530	0.383–0.732	< 0.001
Tirofiban study medication	0.254	0.084–0.766	0.015
Culprit vessel LAD^a^	3.061	1.080–8.677	0.035
Culprit vessel LCx^a^	0.856	0.099–7.413	0.887
TIMI grade flow post PCI < 3	7.444	2.939–18.856	< 0.001
AF on the day of admission	3.585	1.007–12.764	0.049
Zwolle risk score	1.638	1.416–1.894	< 0.001
*Multivariate analysis*			
AF on the day of admission	3.537	0.739–16.927	0.114
Zwolle risk score	1.625	1.404–1.880	< 0.001

*p*-value for odds ratio in the prediction of 30-day mortality

*AF* atrial fibrillation, *BMI* body mass index, *MI* myocardial infarction, *PCI* percutaneous coronary intervention, *BP* blood pressure, *TIMI* thrombolysis in myocardial infarction, *LAD* left anterior descending artery, *LCx* left circumflex artery, *OR* odds ratio, *CI* confidence interval

^a^as compared with culprit vessel right coronary artery

## Atrial fibrillation 24–72 h after admission

Mortality in patients in whom AF was detected 24–72 h after admission was significantly higher compared with patients who were AF-free (14.3 vs 1.4 %, *p* < 0.001), as displayed in Table 1. In univariate analysis, age (OR 1.111, *p* < 0.001), diabetes (OR 5.911, *p* = 0.001), Killip class > 1 (OR 7.275, *p* < 0.001), previous myocardial infarction (OR 4.300, *p* = 0.015), systolic (OR 0.708, *p* = 0.005) and diastolic blood pressure on admission (OR 0.611, *p* = 0.009), tirofiban study medication (OR 0.254, P = 0.035), culprit vessel (OR 4.317, *p* = 0.026), TIMI grade flow < 3 post PPCI (OR 4.061, *p* = 0.019) and AF 24–72 h after admission (OR 11.515, *p* = 0.003) were significantly associated with 30-day mortality. In multivariate analysis, AF 24–72 h after admission was independently associated with 30-day post-infarction mortality (adjusted OR 13.476, *p* = 0.006). The analysis is displayed in Table 3.

**Table 3:** Odds ratio analysis for all-cause 30-day mortality in patients with AF detected > 24 h after admission

Univariate	OR	95 % CI	*p*-value
Age (per year)	1.111	1.048–1.177	< 0.001
Gender male	2.146	0.754–6.105	0.152
BMI	1.055	0.913–1.219	0.468
Current smoker	0.375	0.118–1.186	0.095
Diabetes	5.911	2.052–17.025	0.001
Hypertension	1.800	0.646–5.015	0.261
Hypercholesterolaemia	1.044	0.329–3.314	0.942
Killip class > 1	7.275	2.574–20.556	< 0.001
Previous MI	4.300	1.331–13.895	0.015
Previous PCI	0.792	0.103–6.117	0.823
Systolic BP (per 10 mmHg)	0.708	0.555–0.902	0.005
Diastolic BP (per 10 mmHg)	0.611	0.423–0.883	0.009
Tirofiban study medication	0.254	0.071–0.906	0.035
Culprit vessel LAD^a^	4.317	1.194–15.606	0.026
Culprit vessel LCX^a^			N/A
TIMI grade flow post PCI < 3	4.061	1.258–13.106	0.019
AF 24–72 h after admission	11.515	2.300–57.662	0.003
Zwolle risk score	1.564	1.345–1.818	< 0.001
Multivariate analysis			
AF 24–72 h after admission	13.476	2.138–84.954	0.006
Zwolle risk score	1.548	1.311–1.828	< 0.001

*p*-value for odds ratio in the prediction of 30-day mortality

*AF* atrial fibrillation, *BMI* body mass index, *MI* myocardial infarction, *PCI* percutaneous coronary intervention, *BP* blood pressure, *TIMI* thrombolysis in myocardial infarction, *LAD* left anterior descending artery, *LCx* left circumflex artery, *OR* odds ratio, *CI* confidence interval

^a^as compared with culprit vessel right coronary artery

## Atrial fibrillation > 72 h after admission

Mortality in patients in whom AF was detected > 72 h after admission did not differ among patients with AF compared with those without AF (0 vs 1.1 %, *p* > 0.999), as displayed in Table 1. In univariate analysis, age (OR 1.106, *p* = 0.002), diabetes (OR 6.333, *p* = 0.002), Killip class > 1 (OR 5.939, *p* = 0.003), previous myocardial infarction (OR 5.913, *p* = 0.005), culprit vessel (OR 5.298, *p* = 0.034) and TIMI grade flow < 3 post PPCI (OR 5.583, *p* = 0.006) were significantly associated with 30-day post-infarction mortality. No multivariate analysis was performed for AF > 72 h after admission. The analysis is displayed in supplemental Table 2.

## Discussion

The current study reports the temporal association between new-onset AF and 30-day post-infarction mortality. AF on the day of admission and AF 24–72 h after admission were significantly associated with 30-day post-infarction mortality. In contrast, patients in whom AF was detected > 72 h after admission did not show an increased post-infarction mortality.

## Predictors of mortality

In previous studies, age, diabetes, hypertension, Killip class, previous myocardial infarction, blood pressure on admission, culprit vessel and TIMI grade flow have been associated with short-term post-infarction mortality [17–20]. Furthermore, smoking was associated with reduced short-term mortality [18]. The present study is in line with these previous studies. Furthermore, many studies have reported the independent association between AF and short- and long-term mortality in both the otherwise healthy population [21, 22] and in STEMI patients [6, 12, 23]. Several factors may predispose to AF in the setting of STEMI. Peri-infarction AF has been associated with an increased left ventricular diastolic dysfunction and end-diastolic pressure, which may increase atrial stretch and atrial pressure [24, 25]. Furthermore, atrial dysfunction, atrial ischaemia, pericarditis, congestive heart failure due to ischaemia and enhanced sympathetic tone may also play an important role in the genesis of post-infarction AF [26–28]. Finally, a previous study has reported that an irregular ventricular rate decreases cardiac output and increased pulmonary capillary wedge pressure and right atrial pressure, independent of heart rate [29].

## Previous studies

Of note, a study [23] comparing prognosis of AF before and after PPCI reported that only AF post-PPCI was associated with an increase in long-term mortality. However, several differences between the present study and this study are apparent: the present study investigated the impact of AF on short-term mortality, whereas the patients in the study by Beukema et al. [23] were followed-up for 481 days post-PPCI. Furthermore, pre-PPCI AF was associated with an increase in mortality, but only after adjustment for several confounders, AF pre-PPCI was not associated with long-term mortality. The authors did not categorise the AF occurrence in the groups specified in the present study. Of note, this study was conducted in patients at a relatively short distance to the PCI centres [30], and therefore these results may not be applicable in countries with a longer ischaemic time.

## Clinical implications

The present study showed a clear temporal association between AF and short-term mortality. More specifically, patients who develop AF on the day of admission and especially 24–72 h after admission should be considered high-risk patients. In contrast, patients who develop AF > 72 h after admission show a mortality rate no greater than other STEMI patients and possibly should not be considered high-risk patients. Although this study does not provide any evidence, underlying pathophysiological mechanisms may be different in the AF subgroups. Hypothetically, patients who develop AF on the day of admission or 24–72 h after admission are haemodynamically compromised, with a lower blood pressure and a higher Killip class, factors contributing to an increased 30-day mortality. In contrast, AF > 72 h after admission may be more closely associated with atrial fibrosis, and thereby not directly impacting 30-day mortality.

## Limitations

Current study is a substudy of the On-TIME II study and therefore, a predefined power assessment was not made for the relation of incident AF and short-term mortality. Only a multivariate analysis with the Zwolle risk score could be performed. The results of the multivariate analysis should be interpreted with caution due to the limited number of endpoints in this study. Although all variables were prospectively registered, the present study is a post-hoc cross-sectional analysis. Future studies with a larger patient population may be necessary to further elucidate the temporal association between AF and mortality. Patients with asymptomatic AF before inclusion in the study may have influenced the data. All patients were kept on telemetry for at least 48 h but thereafter, an ECG was performed once daily. This may have caused several asymptomatic AF episodes not to have been documented, which may have impacted the present study’s outcome. Furthermore, the AF episode in regard to the onset of ischaemia was not registered.

## Conclusion

Post-infarction AF occurring during the first 72 h after admission for STEMI was an important risk factor for mortality. In contrast, AF detected > 72 h after STEMI was not associated with 30-day mortality.

## Electronic supplementary material

(DOCX 17 kb)
