# Supplementary material for: Prognostic significance of incident atrial fibrillation following STEMI depends on the timing of atrial fibrillation
Source: Neth Heart J. 2015 May 29;23(9):430–5. doi: 10.1007/s12471-015-0709-2 (PMC4547948; doi:10.1007/s12471-015-0709-2)
Supplement: Supplementary file 1 — (DOCX 17 kb) [file 12471_2015_709_MOESM1_ESM.docx]

**Supplemental tables**

**Supplemental Table 1** Baseline characteristics

|  | Total (n=830) | 30-day mortality: yes (n=810) | 30-day mortality: no (n=20) | P-value |
| --- | --- | --- | --- | --- |
| **Age (years)** | 62.1±11.6 | 61.8±11.4 | 74.2±10.7 | <0.001 |
| **Male gender** | 631/830 (76.0 %) | 618/810 (76.3 %) | 13/20 (65.0 %) | 0.242 |
| **BMI (kg/m^2^)** | 26.9 ±3.7 | 26.9 ±3.7 | 27.3 ±4.3 | 0.857 |
| **Current smoker** | 402/827 (48.6 %) | 398/808 (49.3 %) | 4/19 (21.1 %) | 0.015 |
| **Diabetes** | 89/829 (10.2 %) | 82/809 (10.1 %) | 7/20 (35.0 %) | <0.001 |
| **Hypertension** | 276/830 (33.3 %) | 265/810 (32.7 %) | 11/20 (55.0 %) | 0.037 |
| **Hypercholesterolaemia** | 214/829 (25.8 %) | 209/809 (25.8 %) | 5/20 (25.0 %) | 0.933 |
| **Killip class >1** | 96/821 (11.7 %) | 86/801 (10.7 %) | 10/20 (50.0 %) | <0.001 |
| **Previous MI** | 68/828 (8.2 %) | 63/808 (7.8 %) | 5/20 (25.0 %) | 0.006 |
| **Previous PCI** | 68/830 (8.2 %) | 67/810 (8.3 %) | 1/20 (5.0 %) | >0.999* |
| **Previous CABG** | 12/830 (1.4 %) | 12/810 (1.5 %) | 0/20 (0 %) | >0.999* |
| **Previous stroke** | 15/830 (1.8 %) | 15/710 (1.9 %) | 0/20 (0 %) | >0.999* |
| **Systolic BP (mmHg)** | 130.9 ±24.1 | 131.4 ±23.7 | 109.1 ±29.1 | <0.001 |
| **Diastolic BP (mmHg)** | 76.6 ±15.0 | 76.9 ±14.8 | 63.3 ±19.1 | 0.001 |
| **Tirofiban study medication** | 406/830 (48.9 %) | 402/810 (49.6 %) | 4/20 (20.0 %) | 0.009 |
| **Culprit vessel** |  |  |  | 0.053 |
| **- RCA** | 390/821 (47.5 %) | 385/802 (48.0 %) | 5/19 (26.3 %) |  |
| **- LAD** | 340/821 (41.4 %) | 327/802 (40.8 %) | 13/19 (68.4 %) |  |
| **- LCx** | 91/821 (11.1 %) | 90/802 (11.2 %) | 1/19 (5.3 %) |  |
| **TIMI grade flow post PCI <3** | 74/823 (9.0 %) | 66/803 (8.2 %) | 8/20 (40.0 %) | <0.001* |

Data are presented as mean ± their SD or fraction and percentages where appropriate. *AF* atrial fibrillation; *BMI* body mass index; *MI* myocardial infarction; *PCI* percutaneous coronary intervention; *CABG* coronary artery bypass grafting; *BP* blood pressure; *RCA* right coronary artery; *LAD* left anterior descending artery; *LCx* left circumflex artery; *TIMI* thrombolysis in myocardial infarction; *CK-MB* creatine kinase, MB isoenzyme. P-value between AF free and incident AF patient groups**.** * Fisher’s exact test.

**Supplemental Table 2** Odds ratio analysis for all-cause 30-day mortality in patients with AF detected >72 h after admission

| **Univariate** | **OR** | **95 % CI** | **P-value** |
| --- | --- | --- | --- |
| **Age (per year)** | 1.106 | 1.038 – 1.179 | 0.002 |
| **Gender male** | 2.299 | 0.721 – 7.327 | 0.159 |
| **BMI** | 1.100 | 0.950 – 1.273 | 0.201 |
| **Current smoker** | 0.343 | 0.092 – 1.278 | 0.111 |
| **Diabetes** | 6.333 | 1.965 – 20.407 | 0.002 |
| **Hypertension** | 2.057 | 0.657 – 6.437 | 0.216 |
| **Hypercholesterolemia** | 0.957 | 0.257 – 3.568 | 0.948 |
| **Killip class >1** | 5.939 | 1.845 – 19.119 | 0.003 |
| **Previous MI** | 5.913 | 1.733– 20.178 | 0.005 |
| **Previous PCI** | 1.008 | 0.128 – 7.928 | 0.994 |
| **Systolic BP (per 10 mmHg)** | 0.815 | 0.630 – 1.054 | 0.119 |
| **Diastolic BP (per 10 mmHg)** | 0.818 | 0.542 – 1.234 | 0.338 |
| **Tirofiban study medication** | 0.203 | 0.044 – 0.932 | 0.040 |
| **Culprit vessel LAD*** | 5.298 | 1.137 – 24.695 | 0.034 |
| **Culprit vessel LCx *** |  |  | N/A |
| **TIMI grade flow post PCI <3** | 5.583 | 1.638– 19.032 | 0.006 |
| **AF >72 h after admission** |  |  | N/A |
| **Zwolle risk score** | 1.560 | 1.326 – 1.836 | <0.001 |

*AF* atrial fibrillation; *BMI* body mass index; *MI* myocardial infarction; *PCI* percutaneous coronary intervention; *BP* blood pressure; *TIMI* thrombolysis in myocardial infarction; *LAD* left anterior descending artery; *LCx* left circumflex artery; *OR* odds ratio; *CI* confidence interval. P-value between for prediction of incident AF, *as compared with culprit vessel right coronary artery
